# Analysis of soft rot *Pectobacteriaceae* population diversity in US potato growing regions between 2015 and 2022

**DOI:** 10.3389/fmicb.2024.1403121

**Published:** 2024-09-16

**Authors:** Xing Ma, Xiuyan Zhang, Paul Stodghill, Renee Rioux, Smita Shrestha, Brooke Babler, Hannah Rivedal, Kenneth Frost, Jianjun Hao, Gary Secor, Bryan Swingle

**Affiliations:** ^1^Plant Pathology and Plant-Microbe Biology Section, School of Integrative Plant Science, Cornell University, Ithaca, NY, United States; ^2^School of Food and Agriculture, University of Maine, Orono, ME, United States; ^3^Emerging Pests and Pathogens Research Unit, United States Department of Agriculture-Agricultural Research Service, Robert W. Holley Center, Ithaca, NY, United States; ^4^Department of Plant Pathology, University of Wisconsin-Madison, Madison, WI, United States; ^5^Wisconsin Seed Potato Certification Program, Department of Plant Pathology, University of Wisconsin-Madison, Middleton, WI, United States; ^6^Forage Seed and Cereal Research Unit, United States Department of Agriculture-Agricultural Research Service, Corvallis, OR, United States; ^7^Department of Botany and Plant Pathology and Hermiston Agricultural Research and Extension Center, Oregon State University, Hermiston, OR, United States; ^8^Department of Plant Pathology, North Dakota State University, Fargo, ND, United States

**Keywords:** soft rot and blackleg disease, *Pectobacteriacea*, *Dickeya*, whole genome sequencing, potato

## Abstract

**Introduction:**

Soft rot *Pectobacteriaceae* (SRP) bacteria are globally dispersed pathogens that cause significant economic loss in potato and other crops. Our understanding of the SRP species diversity has expanded in recent years due to advances and adoption of whole-genome sequence technologies. There are currently 34 recognized SRP species that belong to the *Dickeya* and *Pectobacterium* genera.

**Methods:**

We used whole-genome sequencing based analysis to describe the current distribution and epidemiology of SRP isolated from diseased potato samples obtained from commercial potato cropping systems in the United States. Our primary objectives in the present study were to: (1) identify the species of these SRP isolates recovered from potato samples across 14 states in the US, (2) describe the variation among SRP isolates from various US locations and track their temporal changes, and (3) evaluate the evolutionary relationships among these SRP isolates to deduce their source. We collected 118 SRP strains from diseased potato plants and tubers in 14 states between 2015 and 2022.

**Results:**

We identified three *Dickeya* and eight *Pectobacterium* species from diseased potato samples. *Dickeya dianthicola*, *Pectobacterium parmentieri*, *P. carotovorum*, and *P. versatile* appeared to be the predominant species, constituting 83% of the isolates. Furthermore, all *D. dianthicola* strains studied here as well as 90% of US *D. dianthicola* isolates sequenced to date exhibit significant clonality.

**Discussion:**

The prevalence of this specific group of *D. dianthicola*, temporally and geographically, aligns with the occurrence of blackleg and soft rot outbreaks in the northeastern US after 2014. The genomic diversity observed in *P. parmentieri* implies multiple introductions to the US from at least four distinct sources, earlier than the arrival of the predominant group of *D. dianthicola*. In contrast, *P. carotovorum* and *P. versatile* appear to be widespread, long-term endemic strains in the US.

## Introduction

Soft rot *Pectobacteriaceae* (SRP) bacteria are phytopathogenic bacteria that belong to the family *Pectobacteriaceae* and produce extracellular enzymes that break down plant cell walls and give the bacteria access to nutrients within plant cells. SRP bacteria are rod-shaped, gram-negative, facultatively anaerobic bacteria that were previously classified under the genus *Erwinia.* They were recently reclassified into the genera *Pectobacterium* and *Dickeya* (Hauben et al., 1998; Gardan et al., 2003; Czajkowski et al., 2015) and subdivided into 22 *Pectobacterium* and 12 *Dickeya* species (Home - Taxonomy - NCBI (nih.gov)). This reorganization, including the naming of many new species of SRP has been facilitated by advances in whole genome sequencing-based methods such as average nucleotide identity (ANI) and digital DNA–DNA hybridization (dDDH) (Zhang et al., 2016; Tian et al., 2016; Parkinson et al., 2014; Hugouvieux-Cotte-Pattat et al., 2019; Hugouvieux-Cotte-Pattat and Van Gijsegem, 2021; Pedron et al., 2019; Zhou et al., 2022; Oulghazi et al., 2019; Dees et al., 2017; Pasanen et al., 2020). Solving the nomenclature can help stakeholders understand differences between the SRP groups in regards to the host ranges, virulence variances, and their origins (Toth et al., 2021).

Given their wide host range, most SRP species can infect potato (*Solanum tuberosum*) and cause diseases (Ma et al., 2007; Toth et al., 2011). Potato diseases caused by SRP pathogens are named based on the symptoms and the plant parts affected. Soft rot affects potato tubers, causing the tuber flesh to become macerated to a soft consistency that may have a foul odor and darken when exposed to air. Blackleg occurs in stems during the early stages of growth when inky stem rot occurs at the soil line as SRP cells move from sprouting seed tubers to stems. Aerial stem rot occurs when SRP bacteria enter through the natural openings or wounds and causes rotting symptoms on potato shoots and leaves (Stevenson et al., 2001).

Several factors make diseases caused by SRP difficult to control. There are no commercial potato varieties with high-level resistance, so control is limited to seed sanitation practices, including seed treatment and seed certification programs that help to reduce the pathogen levels in seed materials (Czajkowski et al., 2011). However, SRP bacteria have the ability to cause latent, asymptomatic infections that can prevent seed certification scouts from detecting the SRP-related symptoms and getting an accurate assessment of the levels of disease in the crop. Other supplemental practices include field selection, cultivation management, and optimization of storage conditions (van der Wolf et al., 2021).

The major source of SRP inoculum for potato field infection is believed to be carried by latent infected seed potatoes (Perombelon, 1974; Perombelon and Kelman, 1980; Perombelon, 2002; Czajkowski et al., 2011). These latent infections can remain dormant until environmental cues, such as moisture, temperature, or anaerobic conditions stimulate the bacteria to resume virulent growth. But seed tubers are not the only inoculum sources. Some of the SRP bacteria can become soilborne and survive up to 6 months in ideal soil environments (Czajkowski et al., 2011). SRP bacteria can move in soil to neighboring tubers and roots by ground water (Czajkowski et al., 2010) and can also be carried by aerosols in rain for several hundred meters before deposition (Czajkowski et al., 2011). These abilities enable the transmission of SRP bacteria to other plants in the same or adjacent fields.

Since 2014, widespread outbreaks of blackleg and soft rot diseases on potatoes have been documented in the northeast and midwest US (Jiang et al., 2016; Charkowski, 2018; Ma et al., 2018; Ma et al., 2021b; Cai et al., 2018; Rosenzweig et al., 2016; Curland et al., 2021; Patel et al., 2019). In New York State these diseases have been mainly caused by *P. parmentieri*, *P. versatile*, and *D. dianthicola* (Ma et al., 2018; Ma et al., 2021b). In addition to New York, *D. dianthicola* has been detected in Northeast, Mid-Atlantic, and Midwest of the US as well as in two Canadian provinces (Charkowski, 2018; Jiang et al., 2016; Cai et al., 2018; Rosenzweig et al., 2016). A 2015–2016 investigation on multiple potato growing regions determined that the *D. dianthicola* and *P. parmentieri* are the predominant species among many other SRP species discovered in the US (Curland et al., 2021). In the present study, we sequenced the genomes of 118 SRP isolates between 2015 and 2022 from 14 potato growing states across the US to: (1) Determine the species of these SRP isolates, (2) Understand the genetic variation in SRP isolates from different locations and their changes over time, (3) Assess the evolutionary relationships between these SRP isolates to infer epidemiological insights.

## Materials and methods

### Bacterial strains and culture conditions

Bacterial strains were isolated from field samples originating in 14 states across the US by researchers working in each region (Supplementary Figure S1) using standard methods as described previously (Ma et al., 2021b). These field samples were collected *ad hoc* by growers, extension agents, or researcher from commercial and research plots. Usually, one isolate from each sample was collected except the isolates from Wisconsin samples. Among the Wisconsin samples, strain UW021-1, -3, -4, -5, -6, -7, -8, and -9 were isolated from one stem sample, UW021-11, 17, and 19 were isolated from another stem sample in the same growing season. Bacteria were shipped overnight to Cornell University in stab cultures. The bacteria were temporarily stored at 4°C before they were recovered in Lysogeny Broth (LB) broth (Sambrook et al., 1989; Bertani, 2004) and incubated at 28°C with shaking at 240 RPM overnight for DNA extraction and two copies of each isolate were preserved in 16% glycerol at −80°C.

### Genomic DNA extraction

We extracted bacterial genomic DNA from 1 mL overnight LB cultures using either the phenol-chloroform method (Booher et al., 2015; Ma et al., 2021b), Wizard SV Genomic DNA Purification kit (Toll-Riera et al., 2016) (#A2360, Promega, Madison, WI), or ZymoBiomics DNA Miniprep Kit (#D4300, Zymo Research, Irvine, CA). DNA concentrations were determined with a Nanodrop One at 600 nm (Thermo Fisher, Waltham, MA).

### Whole-genome sequencing

DNA Libraries were prepared by the Institute of Biotechnology at Cornell University from bacterial gDNA using Illumina Nextera DNA prep kits with six PCR cycles. Illumina MiSeq Nano 2×150-bp sequencing was conducted on this library to calculate dilution factors and pooled to ensure that each sample was evenly sequenced. Size selection was used to remove small inserts before genome sequencing.

Whole-genome sequencing for the bacterial isolates was conducted using short-read Illumina platforms in three batches. The New York isolates recovered before 2017 (labeled starting with NY14, NY15, and NY17) were sequenced at Cornell Institute of Biotechnology as detailed in the previous study (Ma et al., 2021b), using the Illumina NextSeq 500 platform with the high-output kit, producing up to 400 M single-end 75-bp reads, which consists of 30 Gbp data (referred to as Sequencing platform A, *n* = 43). The remaining strains, if not specified, were sequenced in the second batch at Azenta Life Science (South Plainfield, NJ) using the Illumina HiSeq platform in 2021. One flowcell can generate up to 2 × 150-bp 350 M paired-end reads, or 105 Gbp data (referred to as Sequencing platform B, *n* = 62). Additionally, isolates (JB133A, JB88B, JB94B, 15A12, 15A54, 16A18, 16A44, 18B42, 18B43, ME175, UMSS2, UMSS4, and UMSS5) were sequenced in a 2022 batch at Cornell Institute of Biotechnology using the NextSeq 550 platform with mid-output kit producing up to 200 M paired-end 2 × 150-bp reads, equal to 60 Gbp data (referred to as Sequencing platform C thereafter, *n* = 13).

### Genome sequence assembly and quality assessment

Genome sequencing raw reads were demultiplexed by the sequencing facilities. We downloaded them to BioHPC servers at Cornell University and checked MD5 hashtags for integrity. The single-end 75 bp reads were cleaned and assembled as described previously (Ma et al., 2021b). The paired end 2×150 bp reads were analyzed using a customized pipeline^1^ to clean and assemble reads. This pipeline containerized Unicycler v0.5.0 and other applications to streamline the assembly procedure. In the pipeline, the raw reads were trimmed to remove adaptors and low-quality reads with fastp v0.23.4 (Chen et al., 2018). The trimmed reads were processed by Unicycler v0.5.0 (Wick et al., 2017) to build draft assemblies. The draft assemblies were corrected with trimmed Illumina short reads by NextPolish v4.1 (Hu et al., 2020) to generate polished assemblies. The polished assemblies were annotated using Prokka v1.14.5 (Seemann, 2014). We checked the completeness of each genome assembly using BUSCO 5.5.0 (Seppey et al., 2019) in the genome mode with the enterobacterial lineage database (enterobacterales_odb10).

### Resolve species identity with ANI and dDDH

ANI scores were computed by Referenceseeker v1.8.0 with GTDB and RefSeq databases accessed on 3/31/2021 (Schwengers et al., 2019) for the query strain to determine its genus. The query strain was aligned in FastANI v1.33 against a set of reference group strain database (Jain et al., 2018). This customized database consisted of 580 RefSeq SRP bacterial assemblies (Supplementary Table S1). The comparison results were ranked from the high ANI score to low. The reference strains that have higher than 95% similarity were examined to determine the species of the query strain.

We identified one isolate (1950-12), for which taxonomy could not be resolved by ANI analysis (i.e., no ANI score above 95% to any genome sequence in RefSeq database, accessed on June 27, 2022). We used the Type (Strain) Genome Server (TYGS, ggdc.dsmz.de) to calculate the dDDH score for isolate 1950-12 (Meier-Kolthoff et al., 2022). TYGS uses their algorithms to estimate the real DDH scores based on the query and the reference genome sequences. The Formula d_6_ (a.k.a. GGDC Formula 2) was chosen for the dDDH analyses because it is independent of genome length and, therefore, useful for incomplete genome assemblies. Strains above the cut-off of 70% similarity were considered the same species. The dDDH between 1950-12 and *P. aroidearum* L6 (NZ_CP065044.1) was computed with the Genome-to-Genome Distance Calculator.^2^

### Pathogenicity bioassay

We cultured a loopful of bacterial colony in 5 mL of LB broth and incubated overnight at 28°C with shaking at 240 rpm to reach 10^9^ CFU/mL. We made 5 mm deep holes on yellow flesh potato tubers with a 2 mm diameter sterile wooden applicator and placed 10 μL of inoculum in the hole. For mock controls, 10 μL sterile LB was applied. The potatoes were incubated at 28°C overnight. We cut a longitudinal section of tuber through the inoculation points to observe the maceration symptoms. We picked a loopful of macerated tissue and diluted it in 1 mL of sterile water. A new loop was used to streak the resulting solution on CVP medium. Single colonies that can form pits were selected on CVP after 24 h incubation at 28°C. Colony PCR or PCR from bacterial genomic DNA was conducted with primers dnaXf/dnaXr (Slawiak et al., 2009; Ma et al., 2018). The PCR products were sanger sequenced and we compared these sequences with their genome assembly. The bioassays were conducted twice.

### Phylogenetic analysis

The files in GenBank format for each bacterial genome assembly were generated by the Prokka v1.14.5 annotation tool in the customized pipeline previously mentioned. Additionally, we downloaded the GenBank formatted files (with suffix “.gbff”) for each reference genome of *Pectobacterium* and *Dickeya* species in the RefSeq database on NCBI websites (Genome - NCBI - NLM (nih.gov)). These files were used as input files in a get_homologues pipeline (Vinuesa et al., 2018) with the “-t all -e -D -X” arguments to call single copy orthologous gene clusters, where, argument “-t all” specified the gene must present in all taxa, argument “-e” makes the search only find single-copy genes, “-D” enables PFAM-domain scanning, “-X” uses DIAMOND (Buchfink et al., 2015) instead of the regular BLASTP in protein searches for faster aligning speed. The get_homologues pipeline used bidirectional best-hits algorithms to find single-copy homolog genes and compile them into gene clustering files. These files were subsequently processed with the get_phylomarkers pipeline v2.2.6 (Vinuesa et al., 2018). From all the discovered single orthologue genes, get_phylomarkers pipeline can filter the loci by the following three conditions: (1) The genes can pass the Pairwise Homoplasy Index (Phi) tests, indicating a low possibility of recombination; (2) The gene with a gene tree that can pass the kdetree test indicating it does not have an outlier tree topology; (3) The genes or with high Shimodaira-Hasegawa approximate likelihood ratio test branch support values. The remaining genes were concatenated to construct a maximum likelihood tree were constructed by IQ-TREE v2.0 (Nguyen et al., 2015).

Single nucleotide polymorphism (SNP) analyses were performed at the intraspecies level for each of the four species with the most isolates including all published strains from the RefSeq database (accessed on 12/22/2023) on NCBI. Snippy v4.6.0 (Seemann, 2015) was used to call variances compared to the NCBI reference genome of the working species. In the procedure, the application will use as input one reference genome and the query genomes, including the genomes generated in this study and the published genomes of the same species in the NCBI RefSeq database. The query genomes were mapped onto the reference genome, individually. Then, Snippy identified SNPs from the comparison between the query genome and the reference genome. The SNP calling results were combined for all strains under the same species and aligned in full length SNP sequences. The aligned file was analyzed with IQ-TREE v2.2.2.6 to construct a maximum likelihood tree with the “-B 1000” argument for 1,000 replications of ultrafast bootstrapping.

## Results

### *De novo* genome assembly quality assessment

We assembled a collection of 118 SRP isolates from 14 US states that have important roles in the US potato production systems, including seed tuber production and testing, and commercial potato production for fresh market and processing. We purified genomic DNA and submitted samples for library generation and short reads sequencing as described in Materials and Methods. The raw reads from the sequencing facility were used to produce *de novo* contig assemblies for each isolate.

We used sequencing coverage of raw reads, total nucleotide length, BUSCO score, L50, and N50 of the genome assemblies to assess the sequencing depths and assembly qualities. These statistics of sequencing data and assembly quality were documented in Supplementary Tables S2, S3, and described in Supplementary material. Before submitting to GenBank, genome assemblies were filtered to remove sequences shorter than 200 bp. The 15th contig from 1950-32 was split into two contigs to remove adaptor sequence found at positions 343–389 of the original assembly. All assembled and annotated genomes are available at GenBank in NCBI. See Supplementary Table S3 for accession number for each genome.

### Species identification

In this study, we computed ANI scores based on their *de novo* assembled genomes to classify the 118 SRP isolates from 14 US states at the species level (detailed information in Supplementary Table S4). Most states have fewer than 10 isolates except Wisconsin with 14 isolates and New York with 52 isolates (Table 1). The SRP isolates were classified into 12 species, specifically, nine *Pectobacterium* and three *Dickeya* species based on their ANI results (Table 2)*. P. carotovorum* (19.5% of the total), *P. parmentieri* (24.6%), *P. versatile* (19.5%), and *D. dianthicola* (19.5%) were the most frequently isolated species, which together make up 83.1% of all the isolates recovered in this study (Table 2). We also identified one *P. parvum*, NY1749A, and one *P. aroidearum*, 1950-12. This is likely the first time *P. parvum* and *P. aroidearum* were found associated with disease potato in New York State and Hawaii, respectively.

**Table 1:** Number of isolates from each state.

State	Number
FL	5
HI	8
ID	5
MA	3
MD	4
ME	7
MN	5
ND	3
NY	52
OR	6
PA	1
VA	2
WA	3
WI	14
Total	118

**Table 2:** Number of isolates of each species.

Species	Number of isolates	Percent in total number (%)
*Dickeya chrysanthemi*	3	2.5
*D. dadantii*	4	3.4
*D. dianthicola*	23	19.5
*Pectobacterium aroidearum*	1	0.8
*P. atrosepticum*	4	3.4
*P. brasiliense*	6	5.1
*P. carotovorum*	23	19.5
*P. parmentieri*	29	24.6
*P. parvum*	1	0.8
*P.* spp.	1	0.8
*P. versatile*	23	19.5
Total	118	100.0

Only one *Pectobacterium* strain, isolate 1950-15, did not exceed the 95–96% threshold with any published SRP species. The highest ANI scores associated with 1950-15 were obtained from the genomes of *P. aroidearum* MY7 (94.2%) and other ten *P. aroidearum* strains from the RefSeq database, all above 94%, followed by *P. colocasium* (92.3%) and *P. brasiliense* (91.8%). In the dDDH test, the highest dDDH score was from the comparison between the query isolate and *P. aroidearum* L6 with 54.6% similarity followed by *P. colocasium* LJ1 with 46.0% similarity, followed by the score with *P. brasiliense* LMG21371 of 42.7%. None of the dDDH scores were above the 70% threshold for species assignment. These results suggest that isolate 1950-15 cannot be classified into the same species with any published reference strains in the RefSeq database using computational and genomic methods.

**Figure 1:**
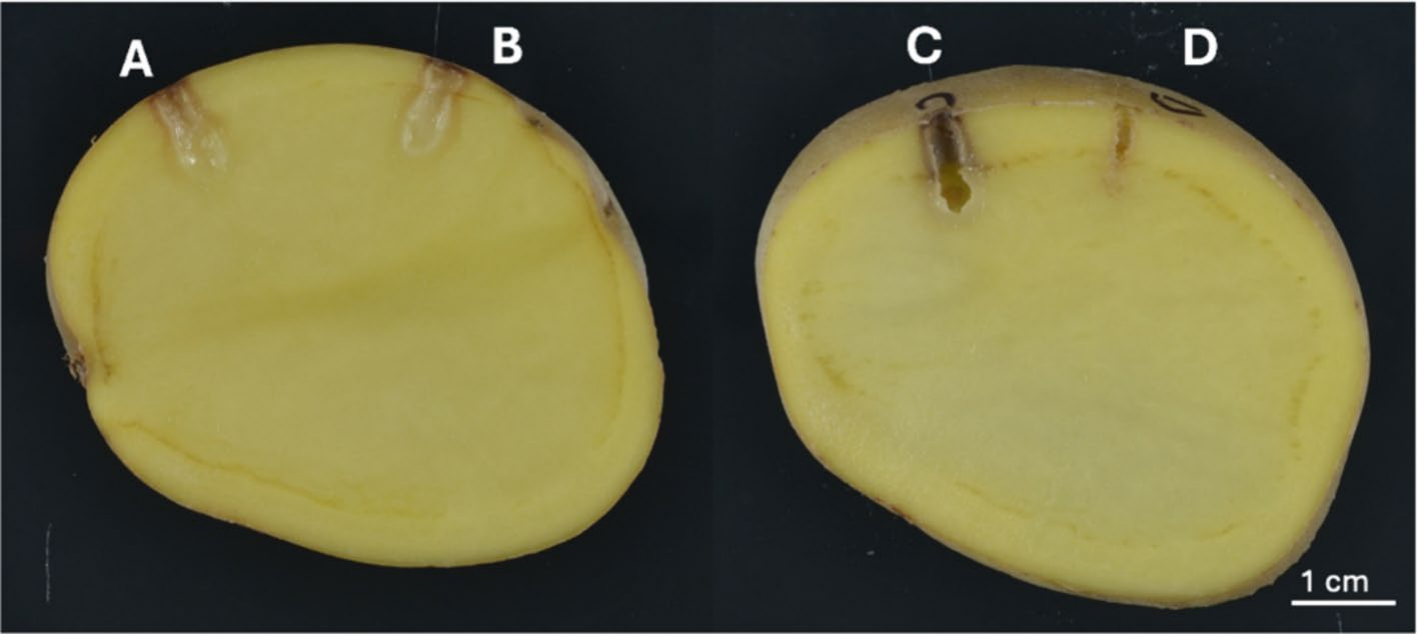
Soft rot symptoms induced in pathogenicity tests on potato tubers. **(A)** *P. aroidearum* 1950-12, **(B)** *Pectobacterium* sp. 1950-15, **(C)** *D. dianthicola* ME23, **(D)** negative control. Scale bar is equal to 1 cm.

The pathogenicity of *P. aroidearum*, 1950-12, and the unknown *Pectobacterium* strain 1950-15 were confirmed with tuber bioassay to complete Koch’s Postulates (Figure 1). Both strains caused extensive macerated tissue surrounding the inoculation points. The same bacteria were reisolated from the macerated tissue and verified by sequencing their partial *dnaX* gene from two replications. No SRP were isolated from negative controls.

### Distribution of SRP bacteria across the US

**Figure 2:**
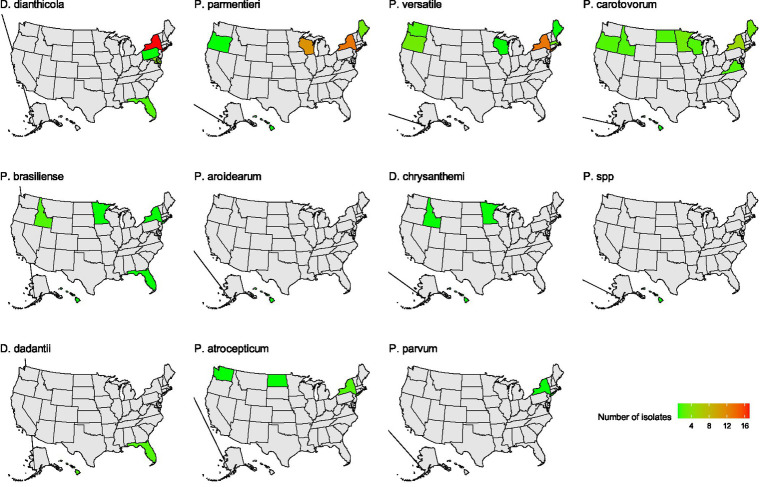
The distribution of soft rot *Pectobacteriaceae* species identified in this study. Green and red color indicates the number of each species identified. Gray color indicates no isolate was obtained from that state.

In this study, *D. dianthicola* was only found in four eastern states of the US: New York, Pennsylvania, Maryland, and Florida (Figure 2). *P. parmentieri* was identified in samples from Maine, New York, Wisconsin, Oregon, and Hawaii. Of these states, New York and Wisconsin had relatively higher numbers of *P. parmentieri* isolates. *P. versatile* was found in samples from six states: New York, Massachusetts, Maine, Wisconsin, Washington, and Oregon. *P. carotovorum* had the most widespread distribution from nine states: Maine, New York, Maryland, Wisconsin, Minnesota, North Dakota, Idaho, Washington, and Hawaii. Six *P. brasiliense* isolates were from five states: New York, Florida, Wisconsin, Idaho, and Hawaii. We identified four *P. atrosepticum* isolates from New York, North Dakota, and Washington. Three *D. chrysanthemi* are from Wisconsin, Idaho, and Hawaii. We did not isolate *D. chrysanthemi* in any state on the east coast. Four *D. dadantii* were found in Florida and Hawaii. The only *P. aroidearum* was isolated from Hawaii. The only *P. parvum* strain was found in New York, which has not been previously reported there. The strain 1950-15 with unknown species was found in Hawaii (Figure 2).

**Figure 3:**
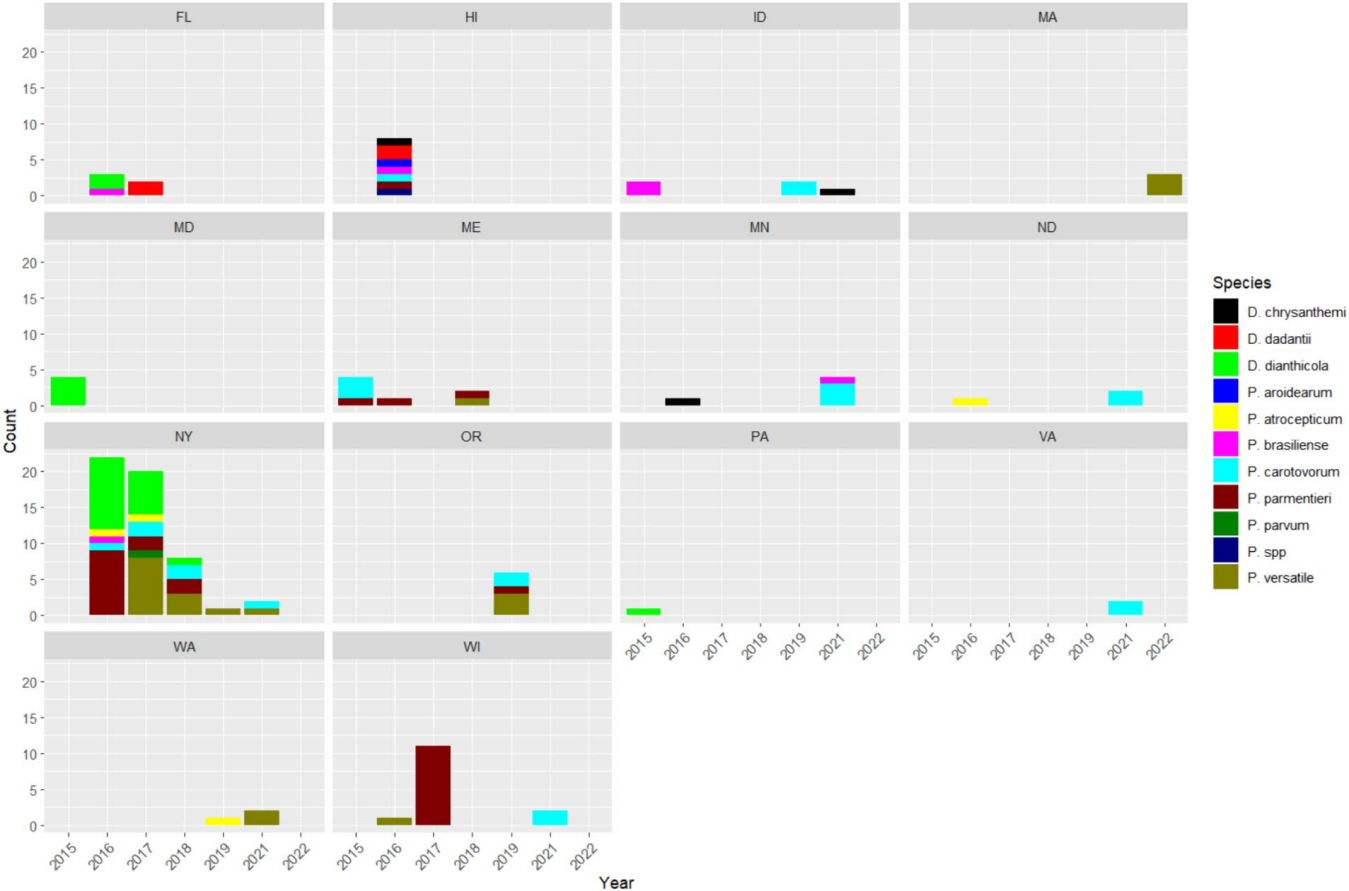
Counts of SRP species identified in each state between 2015 and 2022. The SRP species are represented by different colors.

The number of each SRP species isolated from each state was plotted by year in Figure 3. Our observation of disease and isolation of SRP was sporadic in most states. It is difficult to know why the numbers of SRP varied from year to year, but we suspect that it may be related to several factors, including the incidence of disease in the area, access to fields, awareness of widespread regional outbreaks, and interest in obtaining a diagnosis. For example, there were widespread outbreaks of *D. dianthicola* in the Northeast that began around 2014, this led to an increase in interest in managing this new pathogen and an increase in samples received for diagnosis, particularly in New York. The number of isolates submitted by growers in New York decreased over the 7 year period of this study. The numbers of *D. dianthicola* and *P. parmentieri* isolates detected dropped sharply after 2016 in New York. The decrease in SRP isolates during this time was most likely due to fewer outbreaks caused by *D. dianthicola* in the Northeast and decreasing interest in managing this pathogen. We did not find any *D. dianthicola* after 2018 in all 14 states. *P. versatile* was consistently found in many states since 2018. Many *P. parmentieri* were isolated in 2017 from Wisconsin from two samples. We isolated eight SRP isolates in Hawaii samples in 2016. These Hawaiian isolates are very diverse with at least seven species, namely, *D. chrysanthemi*, *D. dadantii*, *P. aroidearum*, *P. brasiliense*, *P. carotovorum*, *P. parmentieri*, and an undetermined *Pectobacterium* species (Figure 3).

### Phylogeny and epidemiology

**Figure 4:**
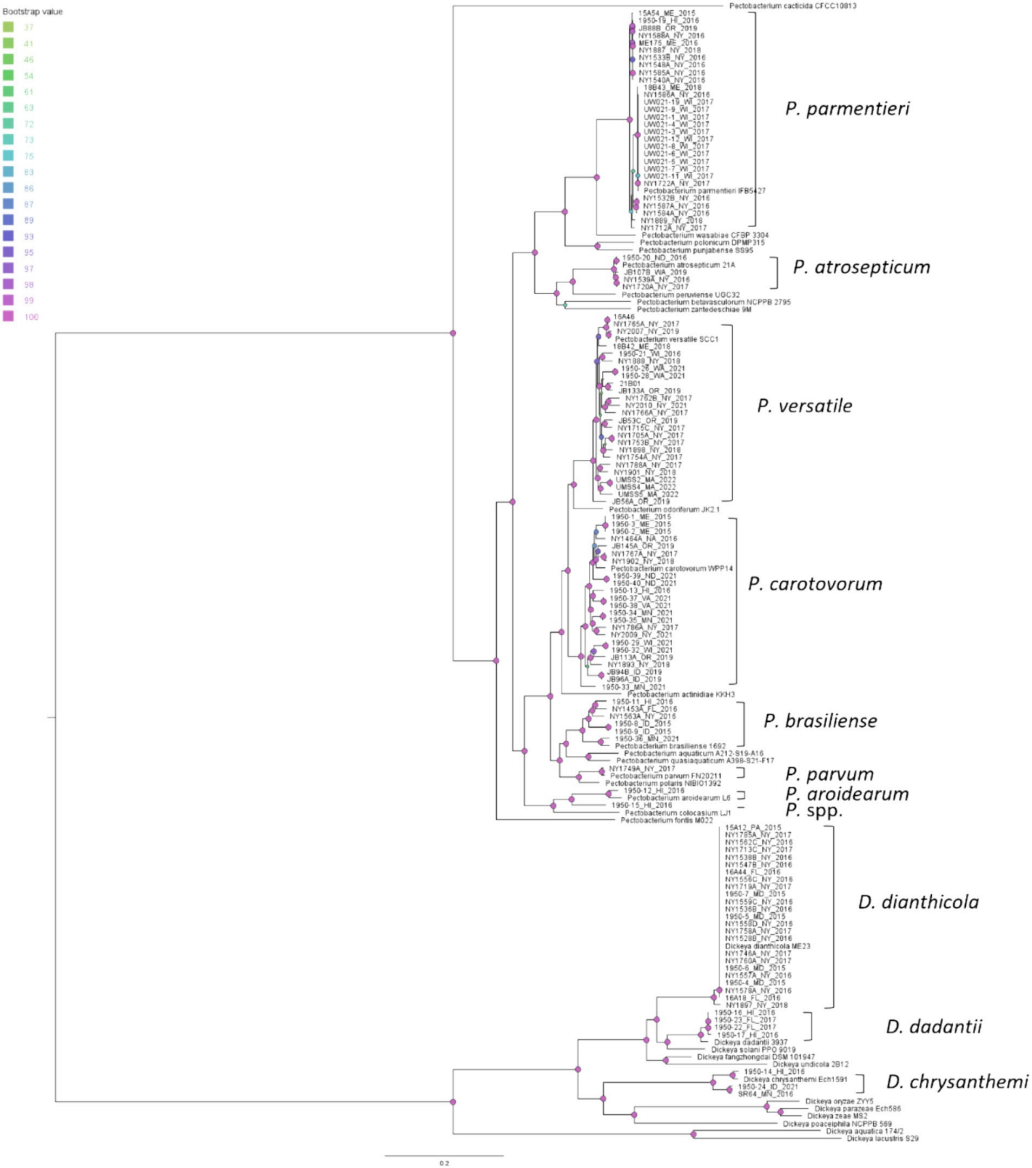
Phylogenetic relationships of soft rot *Pectobacteriaceae* strains from this study and reference strains of *Pectobacterium* and *Dickeya* based on the concatenated 499 single copy orthologue genes shared by the 118 potato isolates, a dahlia isolate NY1897, 21 *Pectobacterium* reference strains, and 12 *Dickeya* reference strains. The query isolates are labeled with strain ID, followed by the state and the year of isolation; the reference strains are labeled with their complete scientific names. Monophyletic clades that contain reference strains and query isolates were labeled with their species names. Bootstrap values were calculated from 1,000 replications of UFBoot2 bootstrapping. The length of the scale bar indicates 0.2 change per nucleotide site.

We constructed a phylogenetic tree to examine the evolutionary relationships among our isolates and reference strains using 499 single copy orthologous genes shared by the isolates in this study and reference strains from the RefSeq database curated by NCBI (Figure 4). The phylogenetic tree separates the *Pectobacterium* and *Dickeya* isolates and reference strains into two distinct clades. The *Dickeya* clade has generally longer branches than the *Pectobacterium* clade, which indicates more evolutionary changes per nucleotide after splitting from their closest common ancestor (Figure 4). The isolates, whose species identified by ANI, always clustered with the reference strain in a monophyletic clade, which agrees with the ANI results (Figure 4).

We conducted SNP analyses for our four most frequently isolated species (*D. dianthicola*, *P. parmentieri*, *P. versatile*, and *P. carotovorum*) to examine the subspecies phylogenetic relationships between our isolates and strains from the RefSeq database. This method was chosen because the accumulation of SNPs among the individuals in these species should produce a very high-resolution phylogeny and allow us to examine the progress of diversification and to test whether we can detect evidence of transmission between potato production regions (Wilson, 2012). Overall, we identified 96,190 SNPs in *D. dianthicola* group, 74,070 SNPs in *P. parmentieri*, 337,091 SNPs in *P. carotovorum*, and 281,515 SNPs in *P. versatile*.

**Figure 5:**
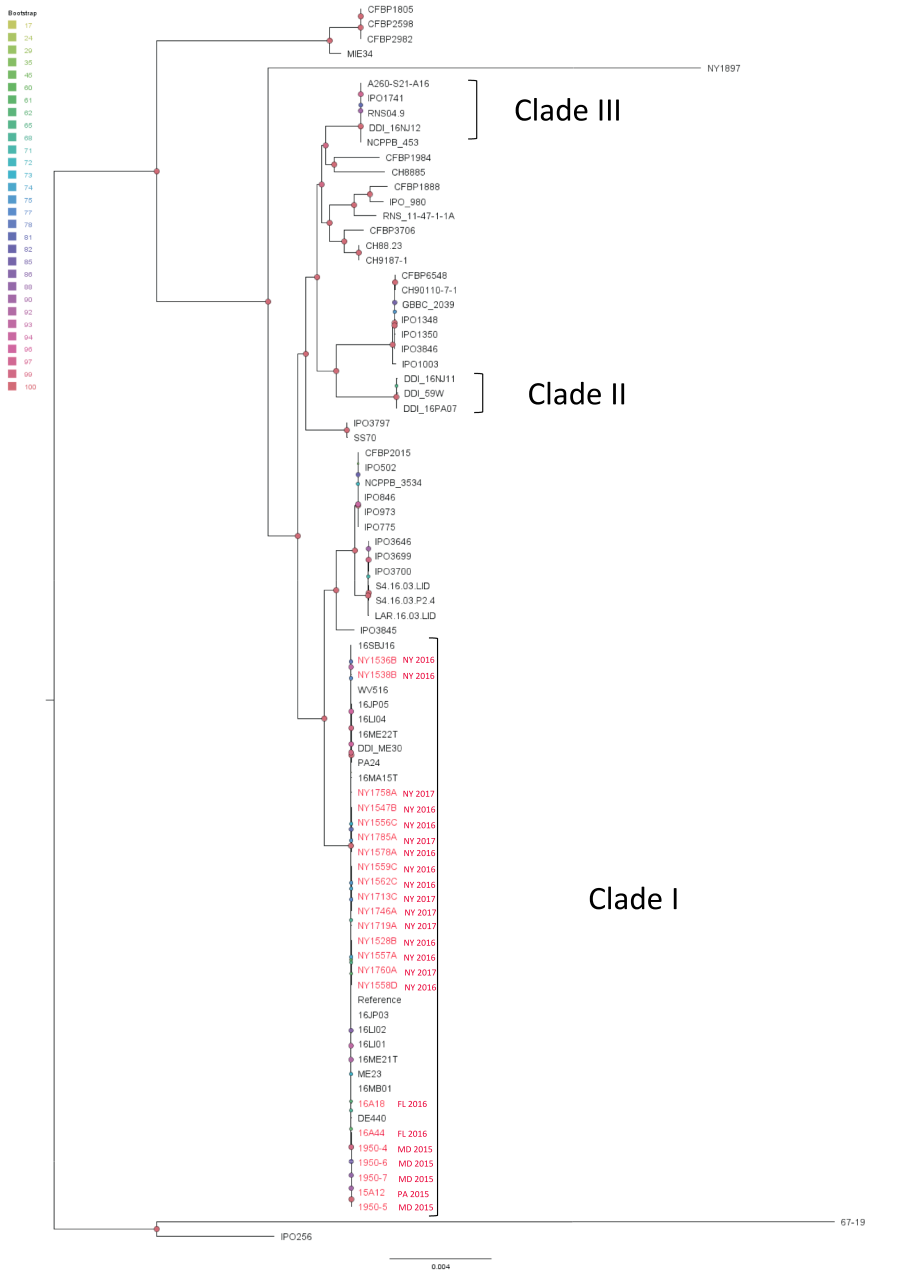
Single nucleotide polymorphism (SNP) based phylogenetic relationship between the *Dickeya dianthicola* isolates from this study (red taxa) and those from the RefSeq database, NCBI (black taxa). The query genomes were compared with the reference genome to identify the SNPs. ME23 was chosen as the reference genome in the SNP analysis. Bootstrap values were calculated from 1,000 replications of UFBoot2 bootstrapping. The length of the scale bar indicates 0.004 change per nucleotide site.

**Figure 6:**
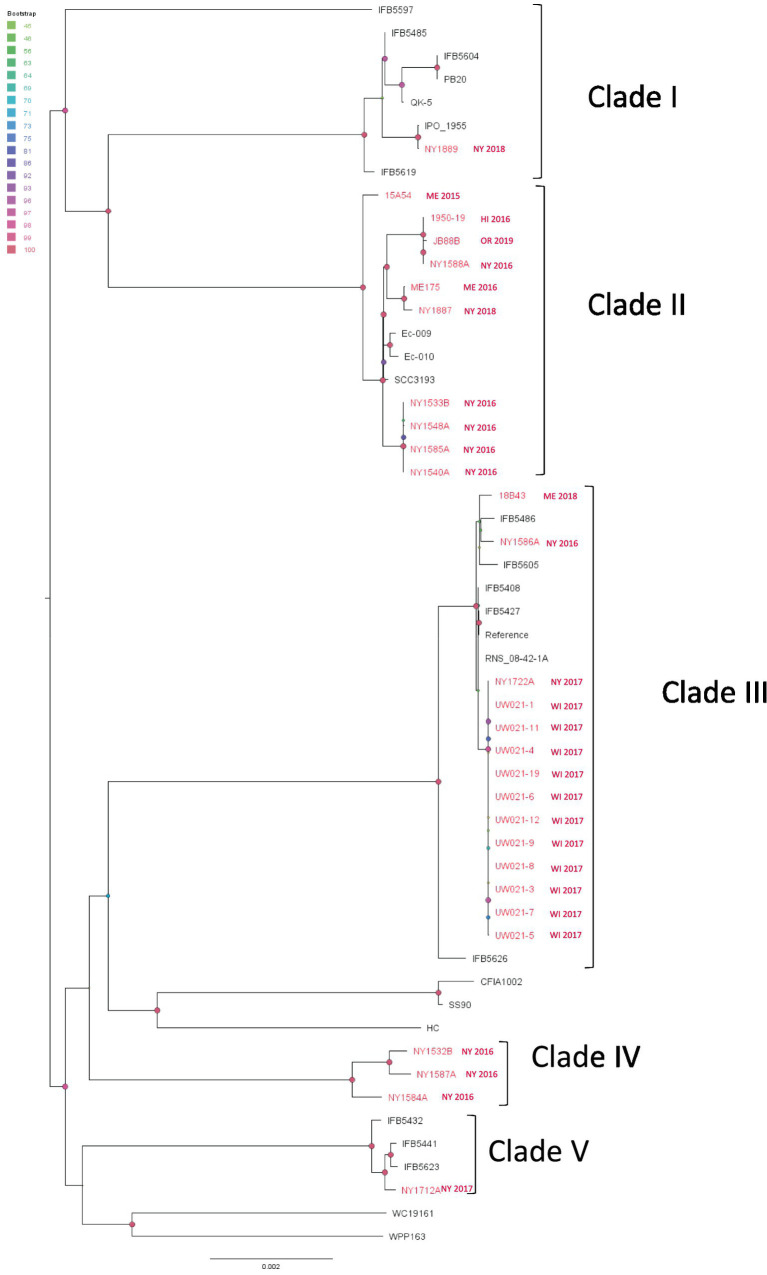
Single nucleotide polymorphism (SNP) based phylogenetic relationship between the *Pectobacterium parmentieri* strains from this study (red taxa) and from the NCBI RefSeq database (black taxa). The query genomes were compared with the reference genome to identify the SNPs. IFB5427 was chosen as the reference in the SNP analysis. Bootstrap values were calculated from 1,000 replications of UFBoot2 bootstrapping. The length of the scale bar indicates 0.002 change per nucleotide site.

All the *D. dianthicola* strains from the present study form a monophyletic clade represented by the reference strain ME23 (Ma et al., 2019) (Figure 5). This clade also includes most northeastern US outbreak strains such as the Delaware strain, DE440, West Virginia strain, WV516, Maine strains ME23, ME30, ME21T, New York strains 16LI01, 16LI02, 16LI04, Virginia strains 16JP03, 16JP05, Massachusetts strain 16MA15T, New Jersey strains 16MB01, Pennsylvania strain PA24 (Ge et al., 2021b) and all sequenced New York isolates (Figure 5). However, a few Northeastern strains are not inside this clade. The New Jersey strain 16NJ11, Pennsylvania strain 16PA07, and a Maine water isolate 59 W formed an independent clade. Another New Jersey strain 16NJ12 groups with some European strains (Figure 6) (Ge et al., 2021b). Interestingly, two non-potato strains from New York, NY1897 (isolated from dahlia) and 67-19 (isolated from impatiens) (Liu et al., 2021), do not belong to any of the three clades (Figure 5).

From the SNP analysis of *P. parmentieri* isolates, we observed that strains in Clade I, II, III, and V were grouped with strains isolated from different continents. And one clade (Clade IV) contains only New York strains (Figure 6). In Clade II, we saw that strains from the east and west coast of the US as well as from New Zealand (Ec-009, Ec-010) and Finland (SCC3193) diverged from a recent ancestor. Note that Clade II isolates from Wisconsin (UW021-XX isolates) were all obtained from only two diseased potato samples (eight from a stem sample and three from a tuber sample). Those Wisconsin isolates are almost clonal along with a New York strain NY1722A. This Wisconsin clade exhibited minimal evolutionary changes from other US and European isolates within Clade II, which implicated a widespread global distribution of *P. parmentieri* strains of this clade. Likewise, in Clade III, strains from New York, Wisconsin, Maine, Belgium (IFB5486), Poland (IFB5605 IFB5608, IFB5427), and France (RNS_08-42-1A) shared a recent common ancestor. Another group that has pandemic features is Clade V, which includes strains from New York State and Poland (IFB5432, IFB5441, IFB5623). Additionally, the 2018 New York strain NY1889 is almost identical to IPO_1995, collected in 2002 with unknown origin (Figure 6). These observations suggest that some strains of *P. parmentieri* were introduced to the US from at least five different sources.

**Figure 7:**
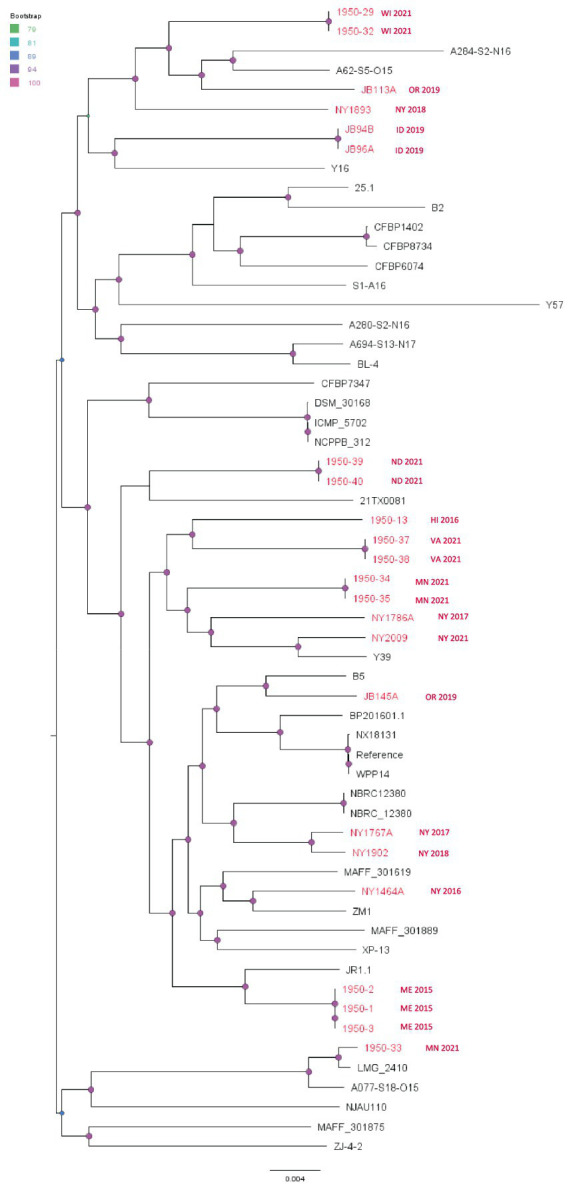
Single nucleotide polymorphism (SNP) based phylogenetic relationship between the *Pectobacterium carotovorum* strains from this study (red taxa) from the RefSeq database, NCBI. The query genomes were compared with the reference genome to identify the SNPs. WPP14 was chosen as the reference in the SNP analysis. Bootstrap values were calculated from 1,000 replications of UFBoot2 bootstrapping. The length of the scale bar indicates 0.004 change per nucleotide site.

The SNP phylogenetic tree of *P. carotovorum* shows that there is more variation among these isolates compared to the *D. dianthicola* and *P. parmentieri* isolates. However, there were six cases of two to three nearly clonal *P. carotovorum* isolates from the same state and same year forming small clusters (Figure 7). The evolutionary changes among those small clusters are significant, as indicated by the long branches between these clusters. This pattern suggests this species has become endemic to many different areas with localized spread within the same year. The isolates from our study (red taxa in Figure 7) did not cluster with any published strains, except 1950-33 (isolated from Minnesota potato in 2021), which has a recent common ancestor with LMG_2410 (isolated from United Kingdom cucumber in 1957) (Figure 7). This finding suggests the intercontinental transmission and a possible host jump might have occurred to this cluster. However, more historic and modern strains are needed to test the hypothesis.

**Figure 8:**
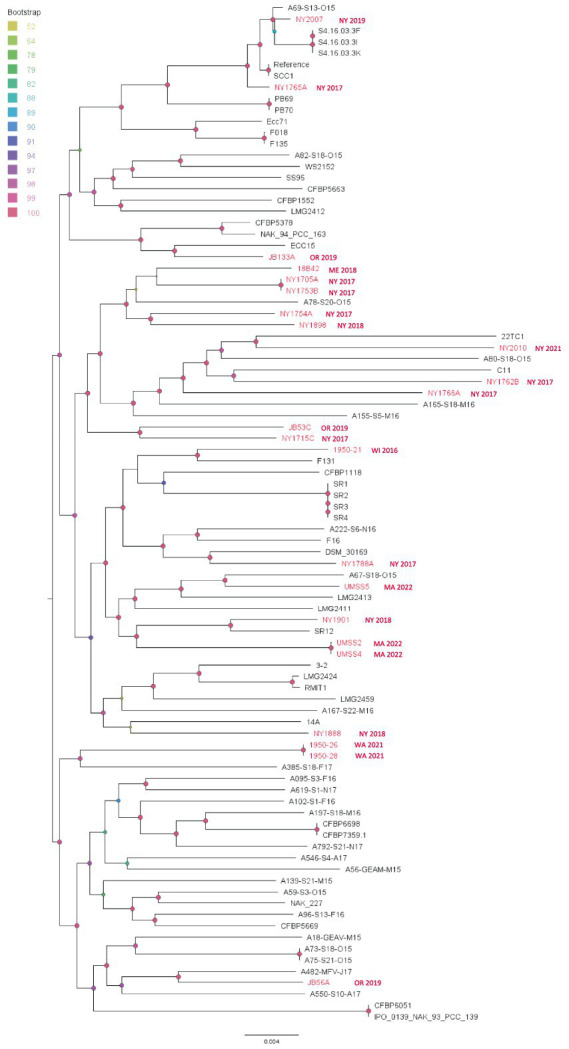
Single nucleotide polymorphism (SNP) based phylogenetic relationships between the *Pectobacterium versatile* strains from this study (red taxa) and from the NCBI RefSeq database, NCBI (black taxa). Bootstrap values were calculated from 1,000 replications of UFBoot2 bootstrapping. The length of the scale bar indicates 0.004 change per nucleotide site. SCC1 was chosen as the reference in the SNP analysis.

The *P. versatile* SNP analysis showed similar patterns as found for *P. carotovorum*. Most of our isolates did not cluster in large clades with recent divergence, but some isolates from the same location and year seem to be clonal (Figure 8). Generally, the branch lengths among different strains are long, which indicates long independent evolutionary histories among those strains. We did not see any strain emerge as a dominant genotype. Therefore, the phylogenetic relationship of *P. versatile* appears to follow a pattern of endemic populations (Figure 8).

## Discussion

In this study we used whole-genome sequencing-based methods to analyze 118 potato SRP strains isolated from 14 states across the US. We identified three *Dickeya* and eight *Pectobacterium* species. Aside from the unidentified species, the rest are known potato pathogens. Koch’s Postulates have been conducted on *P. versatile* isolates from New York, Oregon, and Washington States (Ma et al., 2021a; Ma et al., 2021b). In this study we verified the pathogenicity of the *P. aroidearum* from New York and the unidentified species from Hawaii. Although the bacterial isolates were submitted *ad hoc* by researchers from different states, this study provide a window into the SRP geographic distribution and their changes over time. Whole-genome sequencing based methods maximize the resolution in population diversity and enable us to infer the possible association of SRP species with diseases in different states.

*D. dianthicola* has been found in many northeastern and mid-Atlantic states including Delaware, Maine, Maryland, New Jersey, New York, Pennsylvania, Rhode Island, and Virginia. Other states including North Carolina, Michigan, as well as two provinces in Canada also reported *D. dianthicola* (Ge et al., 2021a; Ma et al., 2021b; Ma et al., 2018; Charkowski, 2018; Cai et al., 2018; Jiang et al., 2016; Patel et al., 2019; Curland et al., 2021). The phylogenetic tree built using 499 single copy orthologous genes showed that the *D. dianthicola* outbreak strains from the US can be classified into three clades, which agrees with the previous SNP analysis (Ge et al., 2021b). Clade I and Clade III in our study correspond to the Sequencing Type 1 (ST1) and ST5, respectively, from a previous analysis using *dnaJ*, *dnaX*, and *gyrB* gene sequences (Curland et al., 2021). But with higher resolution genomic methods, we found that NY1556C assigned to ST2 in the previous study was retained in Clade I. We identified a *D. dianthicola* strain from Florida, which echoes the findings of the 2015–2016 investigation that also identified one *D. dianthicola* in Florida (Curland et al., 2021). To date, *D. dianthicola* appears restricted to the eastern and midwestern US, we did not identify nor has *D. dianthicola* been reported to have caused outbreaks in the Western US potato production areas. We are astonished by the transmission efficiency of the almost clonal Clade I strain in the eastern US, most likely facilitated by seed potato trade networks (Czajkowski et al., 2011). *D. dianthicola* represented by this clade have spread to nine states in the eastern US and we found that about 90% of the sequenced US *D. dianthicola* strains (RefSeq databased accessed on 12/22/2023) and 100% of the *D. dianthicola* isolates that we analyzed in this study are the Clade I strain. This clade does not contain any non-US strains, non-potato strains, or evidence of incremental adaptation from other potato strains. Therefore, its origin remains undetermined. However, the sudden prevalence of this clonal strain in the eastern US coincided with the 2014 outbreaks, strongly suggests its emergence or introduction was a significant factor in causing the widespread outbreaks in the eastern US.

*P. parmentieri* was previously classified under the species *P. wasabiae* and associated with aerial stem rot of potato in Washington State (Dung et al., 2012). Researchers found *P. parmentieri* to be a different species using whole-genome sequence methods (Zhang et al., 2016; Khayi et al., 2016). Later, *P. parmentieri* was recovered from potatoes with soft rot in Minnesota and North Dakota (McNally et al., 2017). The discovery of *P. parmentieri* in the northeastern US also coincided with the blackleg and soft rot outbreaks there (Ge et al., 2018; Ma et al., 2018; Curland et al., 2021). Our results indicate that the introduction of *P. parmentieri* to the US growing fields was probably from at least five sources. The SNP analysis suggests that most of the isolates in each clade are unlikely to be clonal except for some Clade II isolates, but even so, the *P. parmentieri* we isolated across the US have much fewer sequence differences within clades than endemic strains as seen with *P. carotovorum* and *P. versatile*. This finding suggests that their introduction into the US occurred multiple times and probably earlier than the 2014 *Dickeya* outbreaks. But more genome sequences of historic and/or current *P. parmentieri* strains are needed to test this hypothesis.

*P. carotovorum* (previously known as *Erwinia carotovorum*) was one of the earliest described SRP pathogens and, until recently, most SRP isolates were classified as subspecies of *P. carotovorum* (Skerman et al., 1980). Seven new species were later elevated from former *P. carotovorum* subspecies, including *P. atrosepticum*, *P. betavasculorum*, *P. wasabiae*, *P. actinidiae*, *P. carotovorum*, *P. brasiliense*, and *P. oderiferum* (Gardan et al., 2003; Portier et al., 2019). Some species, such as *P. aroidearum* and *P. versatile*, that were previously considered as *P. carotovorum* were elevated to distinct species (Portier et al., 2019; Nabhan et al., 2013). We observed in our study that the modern *P. carotovorum* strains persist in significant numbers, exhibiting widespread distribution in potato-growing states with high genomic diversity. Given the long history and high diversity, *P. carotovorum* is a prevalent endemic pathogen in the US.

Although *P. versatile* was a recently defined species, the earliest known example of a US *P. versatile* strain was isolated from an iris sample 1946 (Portier et al., 2019). Globally, *P. versatile* has been reported from diverse vegetable and ornamental hosts in France, United Kingdom, The Netherlands, Morocco, Algeria, Lebanon, Russia, Serbia, Korea, China, and Canada (Portier et al., 2019; Shirshikov et al., 2018; Marković et al., 2022; Park et al., 2023; Su et al., 2023). In the US, *P. versatile* has been isolated from surface water in Maine and from potatoes in New York, Minnesota, North Dakota, Oregon, and Washington (Ma et al., 2021a; Ma et al., 2021b; Curland et al., 2021). In this study, we isolated *P. versatile* strains from potato in two more states: Maine and Massachusetts. *P. versatile* is one of the most isolated species of SRP across the US potato growing regions. The level of genomic diversity within the species suggests its long-term endemic nature.

*P. brasiliense* appears to have a global distribution. It has been reported from potato tubers in Switzerland, Poland, Russia, Kenya, Turkey, China, and Japan (Onkendi and Moleleki, 2014; Ozturk and Aksoy, 2016; Fujimoto et al., 2017; Voronina et al., 2019; de Werra et al., 2015; Waleron et al., 2015; Zhao et al., 2018). In the US, *P. brasiliense* has been recently reported on potatoes from Minnesota, Pennsylvania, and Hawaii (Boluk et al., 2020; Zhang et al., 2023; Curland et al., 2021). The same species has also been isolated from diseased sugar beets in North Dakota and Minnesota (Secor et al., 2016). Our study discovered that *P. brasiliense* is infecting potatoes in four additional states: New York, Florida, Wisconsin, and Idaho.

Strains of *D. chrysanthemi* were previously isolated in Minnesota (Curland et al., 2021). In addition to Minnesota, we also found strains in Idaho and Hawaii. Compared to Curland and colleague’s study, we did not find any *P. polaris* and *P. punjabense*. But we found a single strain of *P. parvum* and, notably, it is the first time this species was isolated in New York. *P. parvum* is a recently named new species closely related to *P. polaris* (Pasanen et al., 2020). Some of *P. parvum* strains have been in the French Collection for Plant-Associated Bacteria since 1969 (Portier et al., 2020). These SRP strains were only observed in small numbers and their presence fluctuated from year to year.

We reported one strain of *P. aroidearum* from Hawaii. *P. aroidearum* is a pathogen considered to prefer monocotyledon hosts but can also infect dicotyledons (Nabhan et al., 2013). It has been isolated in Asia, South America, and South Africa (Moraes et al., 2017; Li et al., 2022; Tang et al., 2021; Chen et al., 2020; Nabhan et al., 2013). But most of *P. aroidearum* strains were not isolated from potato samples except one report from Lebanon (Moretti et al., 2016). This is the first time that *P. aroidearum* has been found associated with disease in potatoes on US soil and we have conducted pathogenicity tests and complete Koch’s Postulates on this isolate (Figure 1). Interestingly, the *Dickeya* species that cause diseases on monocots (e.i. *D. zeae*, *D. parazeae*, and *D. oryza*) form a clade, which agrees with the previous studies (Yishay et al., 2008).

In conclusion, our study reveals that, across a wide range of US potato growing regions, we identified three *Dickeya* species and eight *Pectobacterium* species in diseased potatoes between 2015 and 2022. Among which, *D. dianthicola*, *P. parmentieri*, *P. carotovorum*, and *P. versatile* were the most prevalent species, making up to 83% of the isolates. All *D. dianthicola* from this study and 90% sequenced US *D. dianthicola* are so far almost clonal. The prevalence of this group of *D. dianthicola* temporally and geographically coincided with the blackleg and soft rot outbreaks between 2014 and 2018 in the northeastern US. The genomic diversity of *P. parmentieri* suggests that some strains were introduced earlier than *D. dianthicola* to the US from at least five different sources. In contrast, *P. carotovorum* and *P. versatile* seem to be widespread endemic species in the US.

## Data Availability

The datasets presented in this study can be found in online repositories. The names of the repository/repositories and accession number(s) can be found in the article/Supplementary material.

## Footnotes

^1^https://github.com/pvstodghill/pipeline-unicycler.git

^2^https://ggdc.dsmz.de
