# Supplementary material for: Analysis of soft rot Pectobacteriaceae population diversity in US potato growing regions between 2015 and 2022
Source: Front Microbiol. 2024 Sep 16;15:1403121. doi: 10.3389/fmicb.2024.1403121 (PMC11439646; doi:10.3389/fmicb.2024.1403121)
Supplement: Supplementary file 1 [file Data_Sheet_1.zip › Supplementary Document.docx]

Supplementary Document

Statistics of whole genome sequencing data and de novo assemblies

Genome assemblies produced by three sequencing platforms exhibited different ranges of sequencing coverage, with 28 – 66X on Platform A, 13-517X on Platform B, and 11- 116X on Platform C (Table S2; Fig. S2A). The genome completeness was assessed by BUSCO. All three platforms have the same median score of 99.6%, specifically, scores ranging from 97.2 – 99.8% for Platform A, 91.8 – 99.8% for Platform B, and 98.9 – 99.6% for Platform C (Table S2, Fig. S2B). The total nucleotide lengths of the assembly from the three platforms ranged 4.6 – 5.3 Mbp (Platform A), 4.3 -5.2 Mbp (Platform B), and 4.7-5.1 Mbp (Platform C), respectively. The three platforms had very close median total nucleotide lengths of 4,865,346 bp (Platform A), 4,855,129 bp (Platform B), and 4,964,440 bp (Platform C), respectively (Table S2, Fig. S2C). We observed that increasing sequencing coverage did not improve BUSCO scores. L50 and N50 are statistics assessing continuity of genome assemblies. L50 is the smallest number of contigs that can cover 50% of the entire genome. N50 is the length of the shortest contig in the L50 set. We found L50 from Platform B has a median of 6, Platform A a median of 22, and Platform C a median of 18 (Table S2; Fig. S2D). The N50 median is 71,860 bp on Platform A, 80,564 bp on Platform B, and 311,117 bp on Platform C (Table S2).
