# Supplementary material for: Analysis of soft rot Pectobacteriaceae population diversity in US potato growing regions between 2015 and 2022
Source: Front Microbiol. 2024 Sep 16;15:1403121. doi: 10.3389/fmicb.2024.1403121 (PMC11439646; doi:10.3389/fmicb.2024.1403121)
Supplement: Supplementary file 1 [file Data_Sheet_1.zip › Supplemental Figures S1 and S2.pptx]

## Slide 1
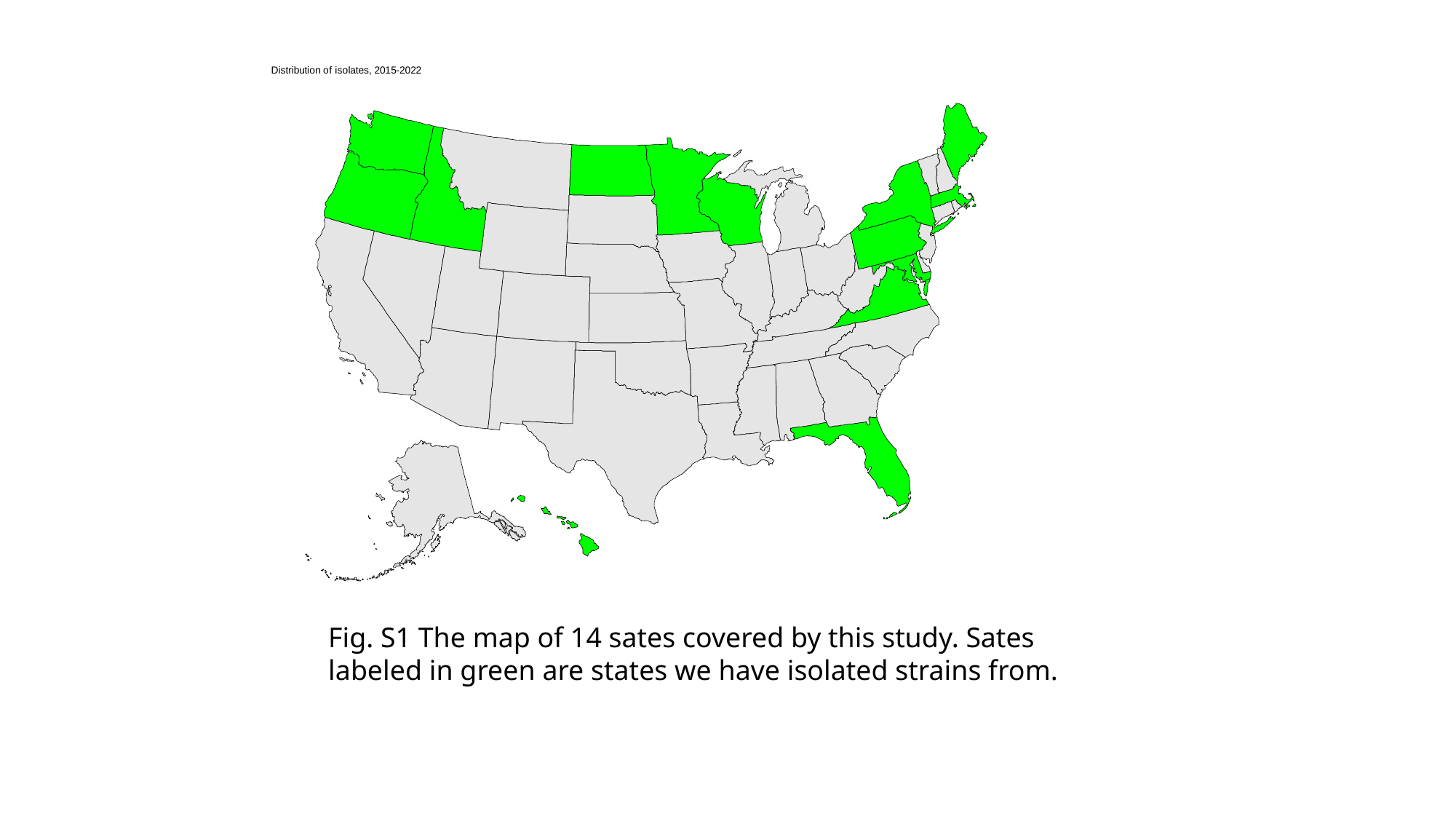

Fig. S1 The map of 14 sates covered by this study. Sates labeled in green are states we have isolated strains from.

## Slide 2
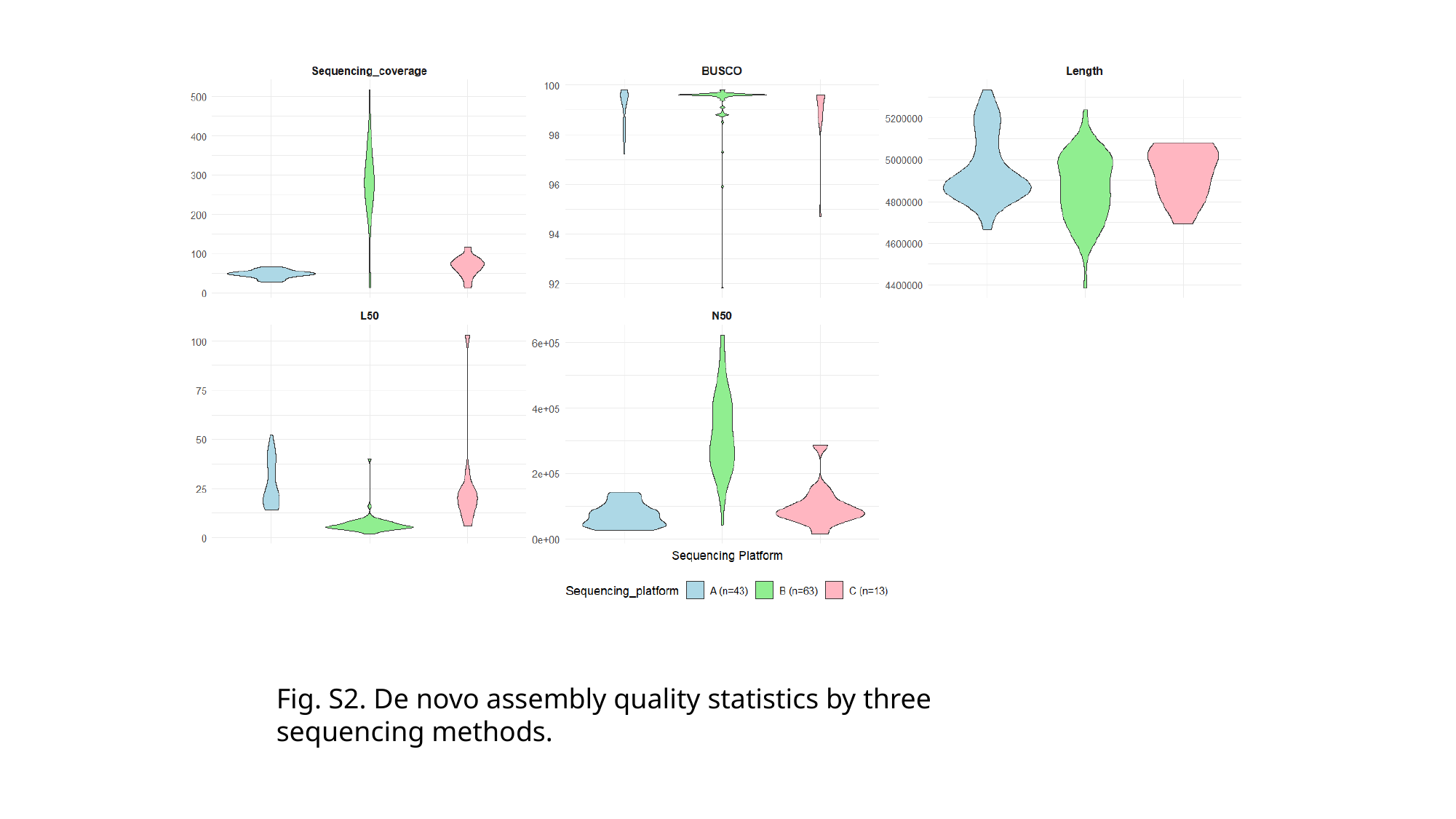

Fig. S2. De novo assembly quality statistics by three sequencing methods.
